# Supplementary material for: Removal of Zinc from Aqueous Solutions Using Lamellar Double Hydroxide Materials Impregnated with Cyanex 272: Characterization and Sorption Studies
Source: Molecules. 2020 Mar 11;25(6):1263. doi: 10.3390/molecules25061263 (PMC7143972; doi:10.3390/molecules25061263)
Supplement: Supplementary file 1 [file molecules-25-01263-s001.pdf]

Supplementary materials:

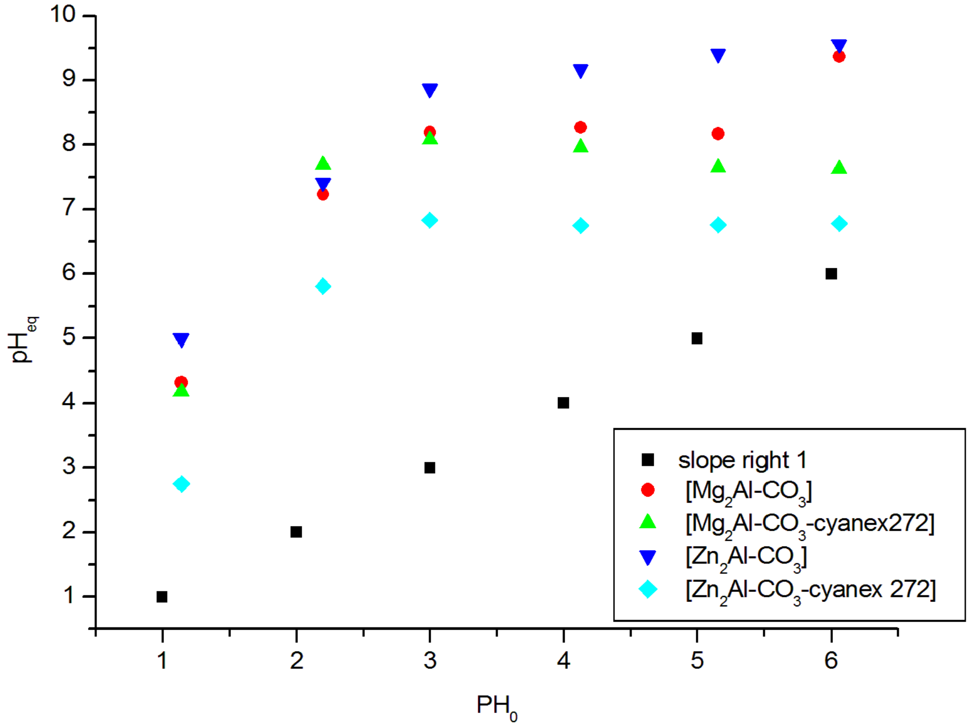

Figure S1. Variation in pH using the impregnated and non-impregnated LDH materials for zinc removal from aqueous solutions
